# Persons living with HIV in sero-discordant partnerships experience improved HIV care engagement compared with persons living with HIV in sero-concordant partnerships: a cross-sectional analysis of four African countries

**DOI:** 10.1186/s12981-021-00363-x

**Published:** 2021-07-22

**Authors:** Domonique M. Reed, Allahna L. Esber, Trevor A. Crowell, Kavitha Ganesan, Hannah Kibuuka, Jonah Maswai, John Owuoth, Emmanuel Bahemana, Michael Iroezindu, Julie A. Ake, Christina S. Polyak, The AFRICOS Study Team

**Affiliations:** 1 Department of Epidemiology, Mailman School of Public Health, Columbia University, 722 W 168th St, Suite 700, New York, NY 10032 USA; 2 U.S. Military HIV Research Program, Walter Reed Army Institute of Research, Silver Spring, New York, MD USA; 3 Henry M. Jackson Foundation for the Advancement of Military Medicine, Inc, Bethesda, MD USA; 4 Makerere University-Walter Reed Project, Kampala, Uganda; 5 HJF Medical Research International, Kericho, Kenya; 6 U.S. Army Medical Research Directorate – Africa, Kisumu, Kenya; 7 HJF Medical Research International Kisumu, Kisumu, Kenya; 8 HJF Medical Research International, Mbeya, Tanzania; 9 HJF Medical Research International, Abuja, Nigeria

**Keywords:** Sero-discordant relationship, Viral load, ART uptake, HIV care continuum, Sub-Saharan Africa

## Abstract

**Background:**

Persons living with HIV (PLWH) who are members of sero-discordant and sero-concordant relationships may experience psychological stressors or motivators that affect HIV care. We assessed the association between sero-discordance status, antiretroviral therapy (ART) uptake, and viral suppression in the African Cohort Study (AFRICOS).

**Methods:**

AFRICOS enrolls PLWH and HIV-uninfected individuals at 12 sites in Uganda, Kenya, Tanzania, and Nigeria**.** At enrollment, we determined ART use through self-report. Viral suppression was defined as HIV RNA < 1000 copies/mL. We analyzed PLWH who were index participants within two types of sexual dyads: sero-discordant or sero-concordant. Binomial regression models were used to estimate prevalence ratios (PRs) and 95% confidence intervals (95% CIs) for factors associated with ART use and viral suppression at study enrollment.

**Results:**

From January 2013 through March 2018, 223 index participants from sero-discordant dyads and 61 from sero-concordant dyads were enrolled. The majority of the indexes were aged 25–34 years (50.2%), female (53.4%), and married (96.5%). Sero-discordant indexes were more likely to disclose their status to partners compared with sero-concordant indexes (96.4% vs. 82.0%, p < 0.001). After adjustment, sero-discordant index participants were more likely to be on ART (aPR 2.8 [95% CI 1.1–6.8]), but no more likely to be virally suppressed. Results may be driven by unique psycho-social factors and global implementation of treatment as prevention.

**Conclusions:**

PLWH in sero-discordant sexual partnerships demonstrated improved uptake of ART compared with those in sero-concordant partnerships. Interventions are needed to increase care engagement by individuals in sero-concordant relationships to improve HIV outcomes.

## Background

With widespread uptake of antiretroviral therapy (ART), persons living with HIV (PLWH) are living longer and more ordinary lives, which includes forming romantic relationships [1, 2]. A sero-discordant couple is a romantic or sexual relationship where one person within the couple is living with HIV and the other person is not. In high prevalence areas like Sub-Saharan Africa, about 50% of PLWH are members of sero-discordant relationships [3, 4]. Transmission of HIV within sero-discordant married or cohabitating couples comprises a significant proportion of new infections in Sub-Saharan Africa and transmission within this key population is an important and preventable driver of the HIV epidemic [5, 6]. Thus, sero-discordant couples present a focal point for HIV prevention and treatment efforts. Landmark studies have established that use of suppressive ART by PLWH is highly effective at preventing transmission to their sexual partners who are at risk for HIV acquisition 7, 8]. However, ART uptake, adherence, and subsequent viral suppression may be influenced by factors such as healthcare access, disease severity, stigma, discrimination, social support, and HIV status disclosure [9–11].

PLWH often experience increased stress and decreased social support due to stigma and societal nonacceptance, which interferes with HIV care engagement and progression through the HIV care continuum, ultimately worsening HIV outcomes [9, 12]. The current body of literature on the impact of sero-discordant partnerships on HIV clinical outcomes has been equivocal. Some studies have found that HIV-infected partners in sero-discordant dyads may experience increased levels of stress due to unique hurdles associated with this type of relationship, like barriers to conception, status disclosure, and stigma that could interfere with ART uptake and adherence, and could result in failure to achieve viral suppression [13–15]. However, other studies have found that sero-discordant dyads may have increased motivation to prevent transmission to their at-risk partner, resulting in greater ART adherence, particularly in more stable partnerships [12, 16]. With the implementation of the U = U (“undetectable [viral load] equals untransmittable”) campaign, further research is needed to elucidate the relationship between sero-discordant partnerships on engagement in HIV care [17]. To add to the current knowledge, among PLWH in sexual partnerships in four African countries, we evaluated associations between HIV sero-discordance status and two key steps in the HIV care continuum: ART uptake and viral suppression.

## Methods and materials

### Study design and participants

Since January 2013, the African Cohort Study (AFRICOS) has prospectively enrolled PLWH and people at heighted risk for HIV at 12 sites in Uganda, Kenya, Tanzania, and Nigeria that are supported by the President’s Emergency Plan for AIDS Relief (PEPFAR) [18]. The South Rift Valley site is comprised of six sites- Kericho District Hospital, Tenwek Mission Hospital, Kapkatet District Hospital, AC LITEIN Mission Hospital, Nandi Hills District Hospital, and Kapsabet District Hospital. The Kisumu West, Kenya site is based in the Kisumu West District Hospital, a Ministry of Health District Hospital in Kombewa, Kenya. The Nigerian sites are located in Abuja and Lagos, Nigeria. The Tanzania AFRICOS site is located at the National Institute for Medical Research-Mbeya Medical Research Center. The Uganda site is housed in the Kayunga District Hospital. PLWH are randomly selected from current clinic patients, new HIV diagnoses, and a group of individuals who have participated in other HIV research studies. People at heighted risk for HIV are recruited from individuals who tested  negative at HIV counseling and testing programs. Additionally, there was particular focus on recruiting sero-discordant partners of PLWH. AFRICOS restricts enrollment to non-pregnant individuals aged 18 years and older.

For this study, we conducted a cross-sectional analysis of enrollment data between 2013 and 2018. At their first visit participants are asked if at least one of their partners are currently enrolled in AFRICOS. For these analyses, participants were considered a member of a sexual dyad if each individual identified the other as a sexual partner on study questionnaires. Sero-discordance status was assessed using HIV test results of the participant rather than self-reported partner HIV status. We categorized dyads as sero-discordant if only one partner was living with HIV and sero-concordant if both partners were living with HIV. In sero-concordant partnerships, the first partner to enroll in the study was considered the index participant and was evaluated for HIV care outcomes. All participants included in this analysis reported being members of a heterosexual partnership.

All participants provided written informed consent for data and specimen collection prior to enrollment. Institutional review boards of the Walter Reed Army Institute of Research, Makerere University School of Public Health, Kenya Medical Research Institute, Tanzania National Institute of Medical Research, and Nigerian Ministry of Defense approved study activities.

### Data collection and outcomes

All AFRICOS participants underwent a thorough medical history, including medical record review and physical examination at enrollment and every six months thereafter. Participants completed broad demographic and behavioral questionnaires at each visit that included a question about whether the participant’s spouse/partner has become aware of his or her HIV status to capture voluntary and non-voluntary disclosure status to their partner. Age, gender, and marital status were ascertained by self-report. Study clinicians classified study participants in one of four ordinal World Health Organization (WHO) clinical stages, where stage 1 is the least severe diagnosis and stage 4 is considered advanced disease [19].

The outcomes of interest were ART use and viral suppression. We defined ART use through self-report (“Are you taking Antiretroviral (ARV) drugs?” [Yes/No]) at enrollment. Additionally, at enrollment PLWH underwent HIV RNA PCR testing using standard clinical assays as previously described [20, 21]. The lower limit of detection varied across sites, ranging from 20 to 48 copies/mL. The WHO defines viral suppression with a value < 1000 copies/mL; however, in most resource-rich settings more conservative goals of < 200 copies/mL and < 50 copies/mL are used due to a growing body of evidence that suggests persistent viremia < 1000 copies/mL increases risk of virologic failure [21, 22]. Due to the variability in viral suppression threshold according to different guidelines, we dichotomized continuous viral load and examined three thresholds of viral suppression (above or below the threshold): < 1000 copies/mL, < 200 copies/mL, and < 50 copies/mL.

### Statistical analysis

Data cleaning was performed in SAS 9.3 (SAS, Cary, NC) and analyses were conducted using Stata 14.0 (Statacorps, College Station, TX). Pearson’s chi-squared and Fisher’s exact tests were used to compare clinical and demographic characteristics of index participants in sero-discordant and sero-concordant sexual dyads. Univariate and multivariate binomial logistic regression models with robust standard errors were used to estimate the adjusted and unadjusted prevalence ratio (PR) and 95% confidence intervals (95% CIs) of the associations of relationship sero-discordance status with [1] ART use and [2] viral suppression. Adjusted PRs (aPRs) controlled for confounders that were selected based on their relevance to sero-discordance and HIV in the literature and were further narrowed by the change in the PR by at least 10% to keep a more parsimonious model. In addition to our primary analysis, we conducted a sensitivity analysis examining the association between sero-discordance status and viral suppression in a restricted subset of individuals on ART for at least 6 months since ART is the main driver for viral suppression.

## Results

From January 2013 to March 2018, 3350 individuals (2790 PLWH and 560 People at risk for HIV) were enrolled in AFRICOS. Of these, 223 were PLWH in sero-discordant dyads and 61 were PLWH in sero-concordant dyads. The majority of the indexes were female (53.4%) and married (96.5%). The overall median age was 39.7 years (interquartile range (IQR): 33.6–46.1); with index participants in sero-discordant partnerships being slightly older than those in sero-concordant partnerships (40.4 [34.1–49.6] vs. 36.3 [31.5–41.8], p = 0.0002; Table 1). As related to partnership dynamics and key HIV indicators, a greater proportion of sero-discordant index partners had partners who were aware of their HIV status (96.4% vs. 82.0% p < 0.0001), had a longer duration on ART (7.8 years [4.8–10.4] vs. 2.8 [1.7–5.7], p < 0.0001), and had more advanced WHO clinical staging (I: 22.4% vs. 45.9%, II: 60.5% vs. 44.3%, III and IV: 13.1% vs. 9.8%, p = 0.0010) compared with sero-concordant index partners.

**Table 1:** Characteristics of PLWH in sexual partnerships in the African Cohort Study

Characteristics	Overall (n = 284)	Index partner in sero-discordant relationship (n = 223)	Index partner in sero-concordant relationship (n = 61)	p-value
Age (years)				
18–24	7 (2.5%)	2 (0.9%)	5 (8.2%)	**0.0002**
25–39	142 (50.0%)	105 (47.1%)	37 (60.7%)	
40–49	77 (27.1%)	62 (27.8%)	15 (24.6%)	
50 +	58 (20.4%)	54 (24.2%)	4 (6.6%)	
Gender				
Man	162 (57.0%)	128 (57.4%)	34 (55.7%)	0.8374
Woman	122 (43.0%)	95 (42.6%)	27 (44.3)	
Marital status				
Single	4 (1.4%)	3 (1.4%)	1 (1.6)	**0.0257**
Married	272 (95.8%)	217 (97.3%)	55 (90.2)	
Divorced/Widowed/Separated/Other	8 (2.8%)	3 (1.3%)	5 (8.2)	
Site				
Uganda	44 (15.5%)	25 (11.2%)	19 (31.2)	** < 0.0001**
Kericho, Kenya	156 (54.9%)	143 (64.1%)	13 (21.3)	
Kisumu, Kenya	31 (10.9%)	20 (9.0%)	11 (18.0)	
Tanzania	26 (9.2%)	18 (8.1%)	8 (13.1)	
Nigeria	27 (9.5%)	17 (7.6%)	10 (16.4)	
Duration on ART				** < 0.0001**
ART Naive	89 (31.3%)	55 (25.7%)	34 (55.7%)	
> 0 months to ≤ 6 months	23 (8.1%)	15 (6.7%)	8 (13.1%)	
> 6 months to ≤ 5 years	101 (35.6%)	87 (39.0%)	14 (23.0%)	
> 5 years	71 (25.0%)	66 (39.6%)	5 (8.2%)	
Year enrolled in cohort				**0.0007**
2013	36 (12.7%)	28 (12.6%)	8 (13.1%)	
2014	123 (43.3%)	109 (48.9%)	14 (23.0%)	
2015	84 (29.6%)	61 (27.4%)	23 (37.7%)	
2016	38 (13.4%)	24 (10.8%)	14 (22.9%)	
2017	3 (1.1%)	1 (0.5%)	2 (3.3%)	
WHO Stage				**0.0010**
I	78 (27.5%)	50 (22.4%)	28 (45.9%)	
II	162 (57.0%)	135 (60.5%)	27 (44.3%)	
III and IV	44 (15.5%)	38 (17.1%)	6 (9.8%)	
Status disclosure to partner				** < 0.0001**
Yes	264 (93.3%)	214 (96.4%)	50 (82.0%)	
No	19 (6.7%)	8 (3.6%)	11 (18.0%)

**Table 2:** Unadjusted and adjusted prevalence ratios and 95% confidence intervals for ART uptake and viral suppression

	ART Uptake		VL < 1000		VL < 200		VL < 50	
	Unadjusted PR (95% CI)	Adjusted PR (95% CI)	Unadjusted PR (95% CI)	Adjusted PR (95% CI)	Unadjusted PR (95% CI)	Adjusted PR (95% CI)	Unadjusted PR (95% CI)	Adjusted PR (95% CI)
Discordance status								
Sero-Discordant	**4.6 (2.5–8.3)**	**2.8 (1.1–6.8)**	**4.4 (2.4–7.9)**	1.5 (0.6–3.9)	**3.9 (2.2–7.1)**	1.4 (0.5–3.4)	**4.4 (2.4–8.1)**	1.9 (0.8–4.5)
Sero-Concordant	Ref	–	–	–	–	–	–	–
Age (years)								
18–24	Ref	–	–	–	–	–	–	–
25–39	1.5 (0.3–7.1)	2.3 (0.3–17.3)	0.7 (0.1–3.6)	0.2 (0.0–2.1)	0.5 (0.1–2.8)	0.2 (0.0–1.5)	0.4 (0.1–2.1)	**0.1 (0.0–0.9)**
40–49	2.4 (0.5–11.8)	2.7 (0.3–23.2)	1.0 (0.2–5.5)	0.3 (0.0–2.8)	0.9 (0.2–5.2)	0.3 (0.0–3.1)	0.8 (0.2–4.6)	0.2 (0.0–2.1)
50 +	**5.5 (1.0–29.7)**	5.0 (0.5–48.6)	2.9 (0.4–18.0)	0.6 (0.1–8.0)	2.2 (0.4–13.0)	0.5 (0.1–7.0)	2.2 (0.4–13.0)	0.5 (0.0–5.2)
Gender								
Man	Ref	–	–	–	–	–	–	–
Woman	**0.6 (0.3–0.9)**	0.9 (0.4–2.1)	**0.5 (0.3–0.8)**	–	**0.5 (0.3–0.8)**	–	**0.5 (0.3–0.8)**	–
Marital status								
Single	Ref	–	–	–	–	–	–	–
Married	8.8 (0.9–86.2)	6.4 (0.3–121.7)	7.6 (0.8–74.2)	–	6.2 (0.6–60.0)	–	5.2 (0.5–51.1)	–
Divorced/Widowed/ Separated/Other	4.0 (0.3–60.3)	21.8 (0.7–654.7)	3.0 (0.2–42.6)	–	3.0 (0.2–42.5)	–	1.8 (0.1–26.2)	–
Site								
Uganda	Ref	–	–	–	–	–	–	–
Kericho, Kenya	**8.9 (4.2–19.1)**	**29.4 (6.3–134.6)**	**7.6 (3.6–16.0)**	**3.9 (1.3–11.3)**	**6.0 (2.9–12.4)**	**3.1 (1.1–8.8)**	**5.0 (2.5–10.1)**	2.4 (0.9–6.6)
Kisumu, Kenya	**5.5 (1.9–16.0)**	**32.5 (5.5–192.3)**	**2.8 (1.1–7.2)**	1.3 (0.3–5.2)	2.4 (0.9–6.2)	1.1 (0.3–4.3)	2.3 (0.9–5.6)	1.1 (0.3–4.0)
Tanzania	1.2 (0.5–3.3)	3.6 (0.6–20.3)	1.3 (0.5–3.6)	2.2 (0.5–8.9)	0.6 (0.2–1.6)	0.5 (0.1–2.3)	0.6 (0.2–1.8)	0.7 (0.2–2.9)
Nigeria	1.9 (0.7–5.1)	**13.1 (2.1–79.2)**	1.4 (0.5–3.7)	1.3 (0.3–5.4)	1.2 (0.5–3.2)	1.1 (0.3–4.4)	0.9 (0.4–2.6)	1.0 (0.3–3.7)
Duration on ART								
ART Naive	N/A	–	Ref	–	–	-	–	–
> 0 months to ≤ 6 months	–	–	**10.3 (3.4–31.0)**	**13.3 (3.9–46.0)**	**6.9 (2.6–18.7)**	**10.5 (3.3–33.6)**	**3.6 (1.4–9.5)**	**5.2 (1.7–15.8)**
> 6 months to ≤ 5 years	–	–	**29.3 (12.8–67.5)**	**23.6 (9.6–58.3)**	**27.3 (12.4–60.1)**	**22.4 (9.4–53.4)**	**19.5 (9.4–40.6)**	**16.7 (7.3–38.1)**
> 5 years	–	–	**48.1 (15.8–146.5)**	**27.5 (8.2–92.5)**	**39.9 (15.0–106.1)**	**19.9 (6.8–57.9)**	**36.1 (14.1–92.0)**	**19.5 (6.9–55.0)**
Year enrolled in cohort	–	–						
2013	Ref	–	–	–	–	–	–	–
2014	1.0 (0.4–2.4)	**0.1 (0.0–0.5)**	0.9 (0.4–2.0)	–	0.7 (0.3–1.5)	–	0.9 (0.4–1.9)	–
2015	0.7 (0.3–1.8)	0.2 (0.0–1.1)	0.6 (0.2–1.3)	–	0.4 (0.2–1.0)	–	0.6 (0.2–1.3)	–
2016	1.3 (0.4–3.7)	0.4 (0.1–2.5)	1.5 (0.5–4.5)	–	1.5 (0.5–4.5)	–	1.9 (0.7–5.3)	–
2017	0.3 (0.1–2.7)	0.2 (0.0–4.1)	0.1 (0.0–1.2)	–	0.1 (0.0–1.2)	–	0.2 (0.0–1.8)	–
WHO stage								
I	Ref	–	–	–	–	–	–	–
II	**3.4 (1.9–6.2)**	**3.5 (1.6–7.6)**	**3.0 (1.7–5.3)**	2.0 (0.8–4.6)	**3.0 (1.7–5.3)**	2.1 (0.9–4.9)	**2.9 (1.7–5.0)**	1.6 (0.7–3.5)
III and IV	**19.5 (4.4–86.1)**	**25.4 (4.5–143.2)**	**5.0 (2.0–12.6)**	2.3 (0.6–8.4)	**4.5 (2.0–10.7)**	2.2 (0.7–7.3)	**4.6 (2.0–10.7)**	2.0 (0.6–6.0)
Status disclosure to partner								
Yes	1.6 (0.8–3.4)	1.1 (0.2–7.6)	**3.7 (1.4–9.5)**	–	**3.7 (1.4–9.8)**	–	**4.0 (1.5–10.8)**	–
No	Ref	–	–	–	–	–	–	–

ART Uptake model adjusted for age, gender, marital status, site, year enrolled in cohort, WHO stage, status disclosure to partner

Viral load suppression models adjusted for age, site, duration on ART, WHO stage

P < 0.05 are bolded

**Fig. 1:**
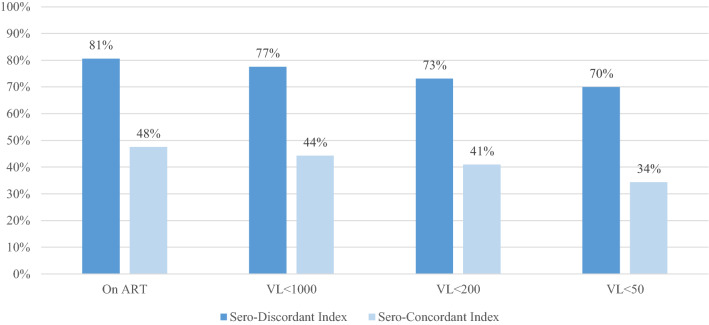
HIV care continuum by sero-discordance status. All chi-squared p-values are < 0.05

At enrollment, 80.6% of sero-discordant index partners were on ART compared with just 47.5% of sero-concordant index partners (p < 0.0001). Additionally, the median viral load was 17 copies/mL (IQR: 1–3350) and a larger proportion of sero-discordant index partners were virally suppressed at all thresholds, 77.1% vs 44.3% with a viral load < 1000 copies/mL, 72.7% vs 41.0% with a viral load < 200 copies/mL, and 69.5% vs 34.4% with a viral load < 50 copies/mL compared with sero-concordant index partners (all p < 0.0001; Fig. 1). After controlling for age, gender, marital status, study site, year of enrollment, there was a 2.8 (95% CI: 1.1–6.8) times increased likelihood of ART use in sero-discordant index participant compared with sero-concordant index participant (Table 2). After adjusting for age, study site, WHO stage, and duration on ART, sero-discordance status was not associated with an increased likelihood of viral suppression at any threshold (< 1000 copies/mL, < 200 copies/mL, and < 50 copies/ml). It is of note, that while status disclosure to partner was not significantly associated with ART use, after adjustment for the confounders listed above, status disclosure was associated with a about a four-fold increase in viral suppression at all three thresholds. For our sensitivity analysis, we examined the association between sero-discordance status and viral suppression among individuals on ART for at least 6 months. In this subset, we found no changes in our conclusion that sero-discordance status was not associated with an increased likelihood of viral suppression.

## Discussion

Using a large cohort study conducted in four Sub-Saharan Africa countries, we evaluated the association between sexual partnership discordance status and two key steps in the HIV care continuum: ART uptake and viral suppression. At cohort enrollment, sero-discordant index partners were significantly more likely to use ART but they were no more likely to be virally suppressed than sero-concordant index partners.

Similar to other studies evaluating serostatus within partnerships and HIV clinical care outcomes, we found that sero-discordant partners have higher ART uptake compared to PLWH in sero-concordant partnerships [12, 16]. The results of a qualitative study conducted in Kenya showed that the majority of PLWH in sero-discordant dyads reported their primary motivation for ART uptake was the prevention of transmission [6]. Another study in South Africa, reported similar motivations and that prevention of transmission may serve as an even greater motivation than individual health benefits [23]. These motivations are often cited in stable sero-discordant dyads, which makes up nearly all sero-discordant dyads in this analysis (97% reported being married). However, these same motivations were not prioritized by individuals without a regular sexual partner [6]. Another study using AFRICOS data assessed the motivations for not using a condom and found that HIV status influences condomless sex and describes trust in a partner as motivation for non-use [24]. Studies that found that PLWH in sero-concordant dyads have better HIV outcomes, reported psychological and social motivations associated with increased social support and companionship, which had an impact on ART use and adherence [13, 16, 25, 26]. The role of psychological and social motivators is key and further research is needed to identify common motivators within each dyad to incorporate those factors into counseling to increase engagement in care.

Over the last two decades, the average time from HIV diagnosis to ART initiation has decreased from approximately two years to only a few weeks among participants enrolled in AFRICOS [27]. This is likely because at the start of the study, ART initiation guidelines were based on CD4 count, which were subsequently updated in 2016 to start all PLWH on ART regardless of CD4 count [28, 29]. Given the differential adoption of these updated guidelines across countries due to delays in implementation and competing treatment priorities, ART data collected at study enrollment may be biased. This is evidenced in another AFRICOS sub study in which prior to 2006, median time to ART initiation ranged from 68 months in Uganda to 4 months in Nigeria. However, by 2016, time to ART initiation was under three months in all countries [27].

Additionally, there may be variability in ART initiation because of study artifact. In 2014, there is an increase in the duration of ART initiation from 2013. When AFFRICOS began enrollment in 2013, participants were recruited from existing clients at PEPFAR clinics. As the study progressed, there was preference in enrolling newly diagnosed individuals who had likely not initiated ART.

Although initiation of ART markedly reduces HIV transmission, residual risk exists in the period before viral suppression is achieved [30]. The critical role of viral suppression in reducing the risk of transmission has become a major focus of campaigns leveraging “Undetectable Equals Untransmittable” messaging, which are particularly relevant to sero-discordant sexual partnerships [17, 31]. We also found that a larger proportion of participants in sero-discordant relationships were virally suppressed at the WHO defined threshold of < 1000 copies/mL [28, 32]. While unlikely, transmission is still possible at this level, [22] as are other adverse HIV outcomes such as progression to viral failure [21]. We therefore evalu ated two additional viral load thresholds, < 200 copies/mL and < 50 copies/mL, which are currently used to define viral suppression in more resource rich countries. These lower thresholds have been shown to be effective in preventing transmission. As such, we found higher proportions of suppressed individuals in sero-discordant relationships compared to sero-concordant ones, however, these associations were likely not powered to determine statistically significant association after controlling for confounding factors. Although sero-discordance status was not significant in our adjusted model, we found that disclosure of HIV status to a partner was strongly associated with viral suppression at all thresholds. While we are unsure if these were voluntary disclosures, other studies have suggested that disclosure is sometimes prompted by a greater desire to mitigate transmission to partner, as well as greater partner support, which is associated with better health outcomes [12, 16]. It is important that interactions with the healthcare system emphasize sustained viral suppression for population and individual health benefits with efforts focused on reaching undetectable viral load levels and status disclosure. Additionally, within partnerships where the PLWH has disclosed their status, achieving viral suppression should be a focus in partner counseling when considering condomless sex within partnerships and supporting engagement in care for improved clinical outcomes.

Our findings should be interpreted in the context of certain limitations. First, we conducted a cross-sectional analysis of baseline data; thus, we cannot infer a causal or temporal relationship between partnership status, ART uptake, and viral suppression. Second, these analyses only included participants who self-reported sexual partnerships with other study participants, which may have biased the population to those in more stable relationships and excluded those with more fluid partnerships that may be impacted differently by psychosocial stressors and motivators associated with sero-discordance or sero-concordance. Participants enrolled in this cohort may not be reflective of the general population, limiting the generalizability of these findings. Further research is needed to examine this association in a population with more diverse sexual relationship statuses, such as individuals with multiple concurrent partners, same-sex partners, and casual partners. Third, although ART status is collected during all participant visits, we used a self-reported ART variable to determine participants’ ART status. This may lead to misclassification of clinical outcomes, although agreement between self-report ART and medical record review is high. Fourth, the number of sero-concordant partners relative to sero-discordant partners was small, which may limit our power to detect a statistical difference in our adjusted analysis that assessed different thresholds of viral suppression. Lastly, based on our bivariate analysis, there were substantial baseline differences between index partners within sero-discordant and sero-concordant partnerships and it is likely that there are additional unmeasured or unknown confounders biasing our results and their interpretation. Despite these limitations, this study showed novel findings in the unique population, quantifying clinical outcomes among PLWH in sero-discordant and -concordant partnerships.

## Conclusions

This study demonstrates that individuals within sero-discordant dyads had better treatment and clinical outcomes compared to sero-concordant dyads. Outside of their own personal benefit, index partners in sero-discordant dyads may be motivated by reducing transmission to their partner in an effort to show commitment. Further research is needed to understand the difference between psychological factors and site level factors that could be in play, such as better establishment of treatment as prevention programs than partner-based HIV treatment.

## Data Availability

The datasets used and/or analysed during the current study are available from the corresponding author on reasonable request.

## Abbreviations

AFRICOS: African Cohort Study
ART: Antiretroviral
CI: Confidence interval
PLWH: People living with HIV
PR: Prevalence ratio
U=U: Undetectable equals untransmittable
